# Vision with different presbyopia corrections simulated with a portable binocular visual simulator

**DOI:** 10.1371/journal.pone.0221144

**Published:** 2019-08-20

**Authors:** Aiswaryah Radhakrishnan, Daniel Pascual, Susana Marcos, Carlos Dorronsoro

**Affiliations:** Laboratory of Visual Optics and Biophotonics, Instituto de Óptica, IO-CSIC, Consejo Superior de Investigaciones Científicas, Madrid, Spain; Nicolaus Copernicus University, POLAND

## Abstract

Presbyopes can choose today among different corrections to provide them with functional vision at far and near, and the outcomes and patient satisfaction depend on the selection. In this study, we present a binocular and portable vision simulator, based on temporal multiplexing of two synchronized tunable lenses allowing see-through and programmable visual simulations of presbyopic corrections. Seventeen binocular corrections were tested: 3 Monofocal (Far, Intermediate, Near), 4 Simultaneous Vision (bifocal, trifocal), 2 Monovision (far and near in either eye) and 8 Modified Monovision corrections (Simultaneous vision in one eye, Monofocal in the other eye). Perceived visual quality was assessed through the simulated corrections in 8 cyclopleged subjects who viewed a composite realistic visual scene with high contrast letters and a landscape at far (4 m) and a high contrast text at intermediate (66 cm) and near (33 cm) distances. Perceptual scores were obtained on a scale of 0 to 5 (low to high perceived quality). Perceptual preference was assessed by judging 36 random image pairs (6 repetitions) viewed through 9 binocular presbyopic corrections using two-interval forced choice procedures. The average score, across far and near distances, was the highest for Monovision (4.4±0.3), followed by Modified Monovision (3.4±0.1), Simultaneous Vision (3.0±0.1) and Monofocal corrections (2.9±0.2). However, the mean difference between far and near was lower for Simultaneous Vision and Monovision (0.4±0.1 PS) than Modified Monovision (1.8±0.7) or monofocal corrections (3.3±1.5). A strong significant correlation was found between the perceptual scores and the percentages of energy in focus, for each correction and distance (R = 0.64, p<0.0001). Multivariate ANOVA revealed significant influence of observation distances (p<10–9) and patients (p = 0.01) on Perceptual Score. In conclusion, we have developed a binocular portable vision simulator that can simulate rapidly and non-invasively different combinations of presbyopic corrections. This tool has applications in systematic clinical evaluation of presbyopia corrections.

## Introduction

Presbyopia is the age-related loss of the ability to dynamically focus near and far [1] and it is usually corrected with an additional positive power (hence called the near addition). The near addition is conventionally provided in the form of spectacles providing alternating vision at far and near. Recently, presbyopia is increasingly corrected with Simultaneous Vision, Monovision and Modified Monovision corrections [2], usually delivered in the form of contact lenses, intraocular lenses, or corneal surgery (such as presbyLASIK or corneal implants). Unlike alternating vision corrections (as progressive spectacle lenses), these solutions are not gaze-dependent and aim at providing clear vision at all distances simultaneously.

Multifocal corrections result in retinal images with superimposed blurred and sharp image components at all viewing distances [2], yet they provide a reasonable visual quality at all distances, while introducing mild degradations at all distances, compared to a single focus [3–5].

While many studies [6, 7] show that subjects generally tolerate simultaneous vision corrections, some patients report [8, 9] functional vision reduction [10] that may even result (in extreme cases) in multifocal IOL explantation or multifocal contact lens wear drop out.

Other popular treatments for presbyopia are Monovision and Modified Monovision corrections [11, 12], where the dominant eye is corrected for far vision and the non-dominant eye is provided with a monofocal correction for near or with a multifocal correction, respectively. Some studies report a reduction in binocular contrast sensitivity and high contrast visual acuity, and stereo acuity in presbyopic subjects with Monovision or Modified Monovision [12, 13].

Previous studies using adaptive optics or on-bench simultaneous vision simulators have allowed testing different multifocal lens designs on the same subject monocularly. We found that the optical design of the multifocal lens is a key factor determining visual performance. On the other hand, strong and consistent inter-subject differences in perceived visual quality also occurred when different subjects judged vision with the same lens design [5, 6, 14–17]. Those differences arose from prior visual experience and, to a large extent, from differences in the optical interactions of the subject’s aberrations and the multifocal lens patterns [14–19]. Besides, the additional need to factor in binocular performance makes it particularly challenging to identify the optimal presbyopic correction for a subject. The ability to customize a presbyopic correction based on the patient’s preference is of interest in the management of presbyopia with contact lenses (as it would allow accelerating lens prescription), and particularly in corrections involving surgery where demonstrating post-operative vision pre-operatively will increase patient’s and surgeon’s confidence in the procedure.

While most vision simulators are primarily experimental on-bench optical setups [15, 16], some systems are making their way into clinical instruments [17, 20]. With the aim of reducing the footprint of typical adaptive optics visual simulators, we developed a monocular two-channel simultaneous vision simulator, with which we demonstrated the effect of near addition on visual acuity [5]. A similar device further incorporating a transmission Spatial Light Modulator was used to compare visual preference across 14 bifocal patterns of different pupillary distributions of optical power (but same energy at far and near), or across orientations in a bifocal segmented rotationally asymmetric design [14–16]. We have recently introduced a portable Simultaneous Vision Simulator [17], with very reduced dimensions thanks to the use of temporal multiplexing, allowing a see-through, large field-of-view and light device. In this system multifocality is achieved by temporal multiplexing of the tunable lenses. In temporal multiplexing, the focus state of the lens is rapidly changed over time, for example between a far focus and a near focus in a bifocal correction. Since the sequence is presented at high speed, the visual system integrates this temporally changing image as a static image containing different foci. The energy at a particular focus is controlled by the amount of time the lens stays in that focus [17], and therefore any thru-focus curve (not only bifocal corrections) can be programmed. Despite the clinical need for testing the presbyopic corrections binocularly, most of binocular visual simulators are restricted to large adaptive-optics based on bench systems, used in the laboratory to study stereopsis or binocular summation under manipulated optics [20–25]

In this study we present a new binocular open-field vision simulator suitable for clinical applications, based on temporal multiplexing, therefore combining the advantages of the portable simultaneous vision simulator and the capabilities of binocular visual simulators. We evaluated perceived image quality in subjects through binocular combinations of monofocal, bifocal, trifocal, Monovision, and Modified Monovision corrections.

## Materials and methods

### Setup: Binocular simulator and visual scene

**Fig 1:**
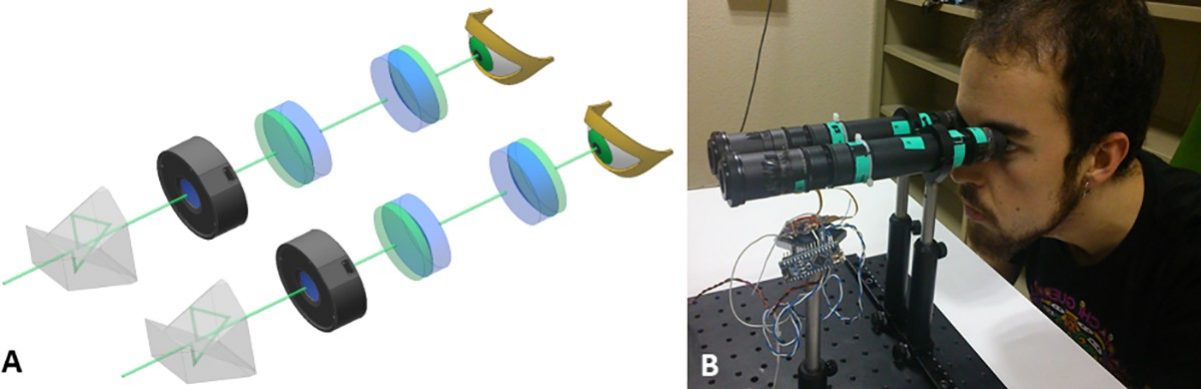
(A) Schematic diagram of the binocular vision simulator. L1 and L2 are projection lenses (50mm EFL) used for optically conjugating the eye and the tunable lens (TL). A Schmidt-Pechan prism is used for image re-inversion (B) Image of a subject viewing through the system. The individual has given a written informed consent (as outlined in PLOS consent form) to publish his photograph.

A binocular vision simulator (Fig 1) was developed with two identical see-through optical channels, which allowed optical induction of monofocal and multifocal corrections. This visual simulator is inspired from a previous monocular version [17], and consists of two rectilinear optical channels, each one with a tunable lens (EL-10-30-C, Optotune Inc, Switzerland), two projection lenses and an erecting prism, mounted on adjustable tube mounts. Both tunable lenses can be controlled independently, and synchronized by a single custom high-speed Arduino-based electronic driver. The tunable lens is optically conjugated with the subjects’ pupil using a pair of achromatic doublets of 50 mm EFL. A Schmidt-Pechan prism is used to render the image erect (horizontally and vertically) and aligned with the line of sight, without affecting the optical quality. However, the use of this prism limited the effective visual field from 20 to 12 degrees. For each channel, a diaphragm placed next to the tunable lens acted as artificial pupil.

The tunable lenses were calibrated and validated by aberrometry, focimetry, and by power matching trial lenses, as described in Dorronsoro et al [17]. The distance between the optical elements was fine-tuned by imaging objects at infinity (>1 km) through the system using a 1-meter collimator on to a CCD camera, ensuring identical focusing and magnification state in each channel. A mechanical rail allows adjusting the interpupillary distance and the horizontal convergence of the channels (while maintaining the vertical co-alignment) to achieve a superposition of the two fields of view compatible with the binocular convergence at far and near distances.

### Visual Scene for Psychophysical task

**Fig 2:**
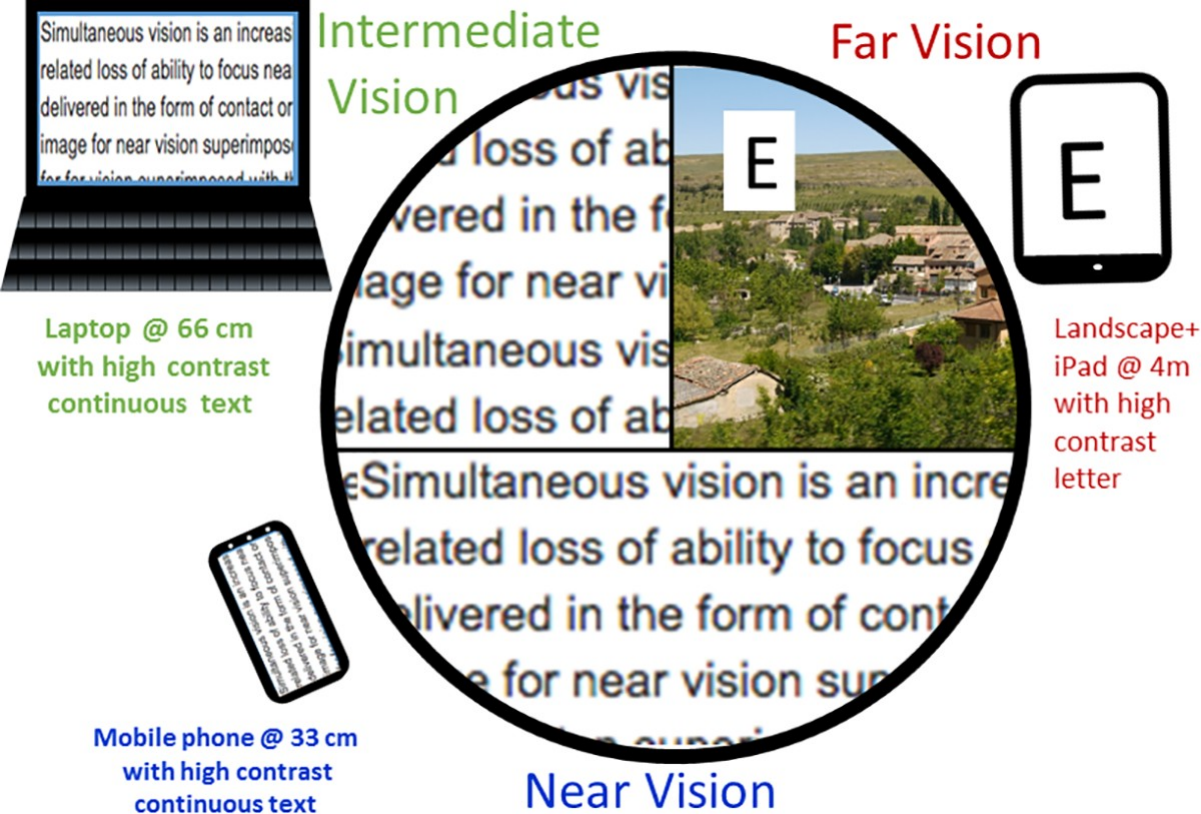
Composite high resolution visual scene for perceptual preference measurements was composed using landscape and high contrast letter (in iPad) for far (4m), and a high contrast text for intermediate distance on a laptop (66 cm) and near distance on a mobile phone (33 cm). The text and the scene is similar but not identical to the original image and is therefore for illustrative purposes only.

Fig 2 illustrates the circular visual scene used in the psychophysical measurements, as seen from the subject’s point of view through the binocular system. Measurements were conducted in indoors ambient light (luminances ~30 cd/m^2^). The scene contains a natural image printed on a poster and a high contrast target on a tablet at far (4 m), text on a laptop at intermediate (66 cm) and text on a smartphone at near (33 cm) distances. The far and intermediate scenes were projected in the upper visual field (60% of the total scene) and the near visual scene was projected in the inferior visual field (40% of the total scene). High definition displays (52 cd/m^2^) were used, with effective angular resolutions higher than the cut-off frequency of the human eye.

### Simulated presbyopic corrections

The perceptual quality and preference was assessed by psychophysical measurements for 17 simulated binocular presbyopic corrections comprising binocularly symmetric (monofocal and simultaneous vision) and asymmetric (Monovision and Modified Monovision) corrections.

For monofocal corrections at far (F+F), intermediate (I+I) and near (N+N), both tunable lenses were placed at a static focus of 0 D (or subjective best focus as determined by the refractive error of the subject), +1.5 D and +3 D, respectively. Binocular simultaneous vision corrections were induced by temporally multiplexing both tunable lenses. We simulated a bifocal correction with 50% of the energy at far and 50% at near (2SV+2SV) and trifocal corrections with 50% at far, 20% at intermediate and 30% at near (3SV+3SV) in both channels, and also combinations of bifocal and trifocal (2SV+3SV, 3SV+2SV) in either of the channels.

Monovision was simulated by focusing one channel at far and the other at near (F+N). Modified Monovision corrections were induced by setting a monofocal focus at far or near in one channel and setting a simultaneous vision correction in the other channel (F+2SV, F+3SV, 2SV+N and 3SV+N). For the Monovision and Modified Monovision, measurements were performed correcting the dominant eye for better far vision (the corrections previously described) but also correcting the non-dominant eye for better vision (labeled as N+F, 2SV+F, 3SV+F, N+2SV, N+3SV).

### Subjects

Eight subjects, with age range 23 to 45 years, participated in the measurements. None of the subjects had astigmatism > 1 D and the spherical refractive error ranged from 0 D to -6 D. All subjects except one (S5) had prior experience in performing psychophysical measurements. Accommodation was paralyzed in both eyes by instilling 3 drops of 1% tropicamide, 15 minutes prior to measurements and hourly to maintain accommodation. The artificial pupil diameter was set to 5 mm to provide uniform pupil size across subjects. Overall, the measurements lasted for about 2 hours.

### Interpupillary distance measurement

For each subject, ocular dominance (using the Miles test) and the refractive corrections (using autorefraction) were assessed. The inter-pupillary distance (IPD) and the horizontal fusional convergence of the binocular simulator were set subjectively, by asking the subject to fuse the targets at each distance. This fusion was checked both pre- and post- cycloplegia and all measurements were performed with this IPD setting.

### Ethics statement

The experiments conformed to the tenets of the Declaration of Helsinki, with protocols approved by the Consejo Superior de Investigaciones Cientificas Ethics Committee. All participants provided written informed consent after the nature and consequences of the study had been explained to them. The individual has given a written informed consent (as outlined in PLOS consent form) to publish his photograph.

### Perceptual scoring measurements

Subjective image quality using a perceptual scoring technique [6] for the 17 binocular presbyopic corrections (F+F, I+I, N+N, 2SV+2SV, 3SV+3SV, 2SV+3SV, 3SV+2SV, F+N, F+2SV, F+3SV, 2SV+N, 3SV+N, N+F, 2SV+F, 3SV+F, N+2SV, N+3SV). The subject viewed the visual scene through each optical correction (which were pre-programmed in the device and presented in random order). Subjects scored the perceived quality of the visual scene from blurred (score 0) to sharp (score 5). Scores were provided for vision at far, intermediate, and near distances, and also for the global visual scene. The measurements were repeated three times and the average score was calculated for each correction.

### Perceptual preference measurements

The preference for a specific presbyopic correction combination was tested using a two-alternative forced choice. Subjects viewed the visual scene for 5 seconds through a presbyopic correction and then through another presbyopic correction for another 5 seconds, and then selected one of the two corrections. In these preference measurements, the dominant eye was always corrected for far. A total of 36 pairs of 9 presbyopic corrections (3SV+3SV, 2SV+2SV, 2SV+3SV, 3SV+2SV, F+N, F+2SV, F+3SV, 2SV+N, 3SV+N) were compared. The measurements were randomized, with 6 repetitions of each pair. The cumulative positive responses were obtained for each correction. Using a Bernoulli cumulative distribution function (significance level on 0.07) a correction with 5 or 6 positive responses was identified as significantly preferred and 0 or 1 as significantly rejected.

## Results

### Perceptual scoring of binocular presbyopia corrections

**Fig 3:**
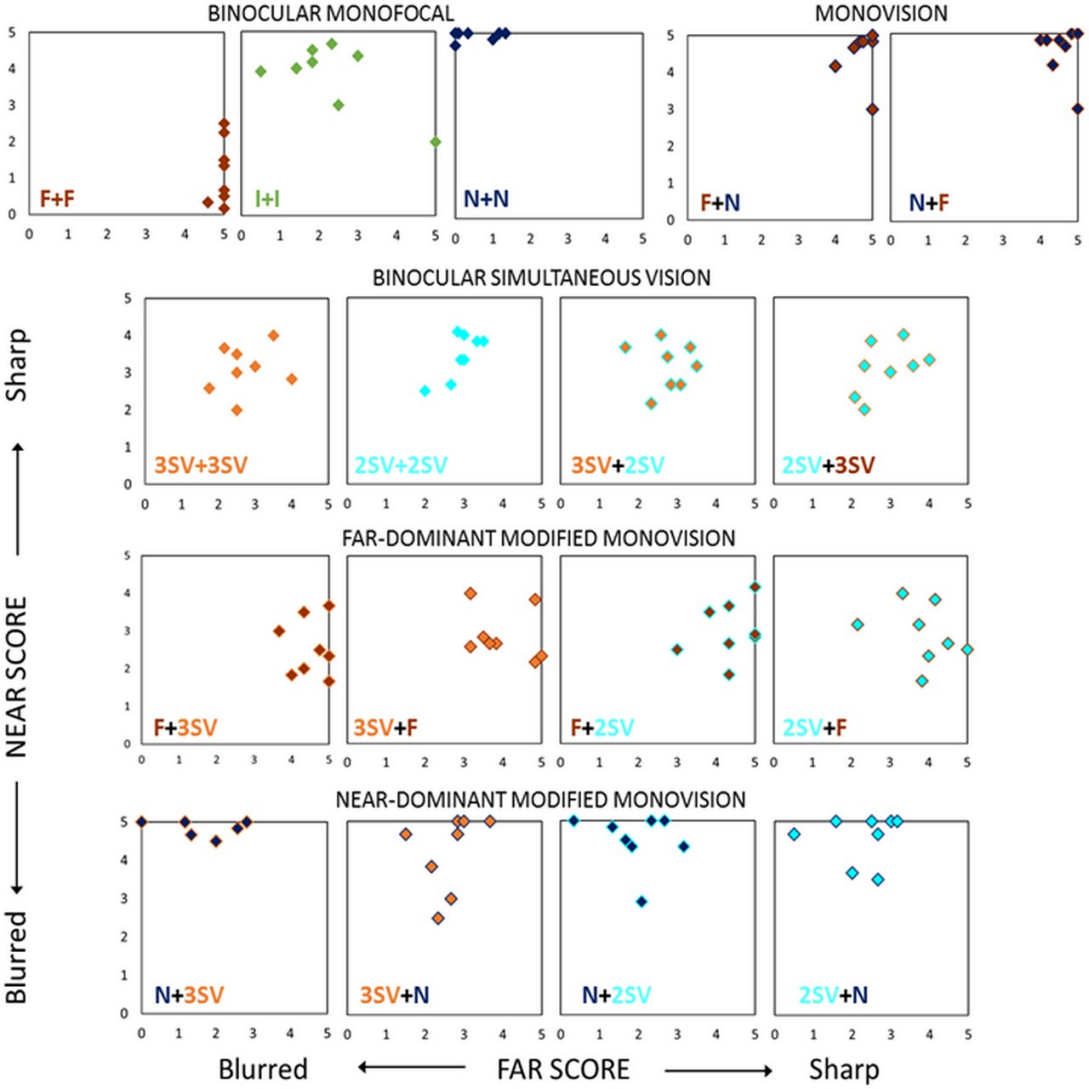
Binocular perceptual score for far (x-axis) vs near (y-axis) for each correction (each box) and each subject (each symbol).

Fig 3 shows the perceptual scores (PS) for all subjects (each symbol represents the average of the three repetitions per subject) and all corrections, for vision at far (horizontal axis) and near (vertical axis) distances. Distinct trends were observed for the different corrections: Monofocal corrections at far (F+F) or near (N+N) produced best perceptual quality (> 4.7 PS) but only at far or at near distances (in-focus distances) creating the expected imbalance between far and near vision. The scores for intermediate monofocal correction (I+I) varied widely across subjects. The mean difference in the perceptual score between far and near was lower for Binocular Simultaneous Vision and Monovision (0.4±0.1 PS) than Modified Monovision (1.8±0.7 PS) or monofocal corrections (3.3±1.5 PS). The average score across far and near distances was the highest for Monovision (4.4±0.3 PS), followed by Modified Monovision (3.4±0.1 PS), Binocular Simultaneous Vision (3.0±0.1 PS) and Monofocal corrections (2.9±0.2 PS). These results suggest that even though monofocal corrections produce better perceptual quality when in-focus, simultaneous vision and Monovision corrections provide a more homogeneous vision across near and far distances. It can also be noted from Fig 3, that with Modified Monovision, as well as with F+F or N+N, the percentage of energy for far and near determines the balance between far and near in vision. The PS at a specific distance correlated significantly (p<0.0001) with the energy provided by the correction at the corresponding distance, averaged between both eyes (r = 0.65), with the energy provided by the correction at better focus for that distance in either eye (r = 0.66) or with the energy provided by the correction for that distance in the dominant eye (r = 0.59).

### Perceptual scoring and subjective differences

**Fig 4:**
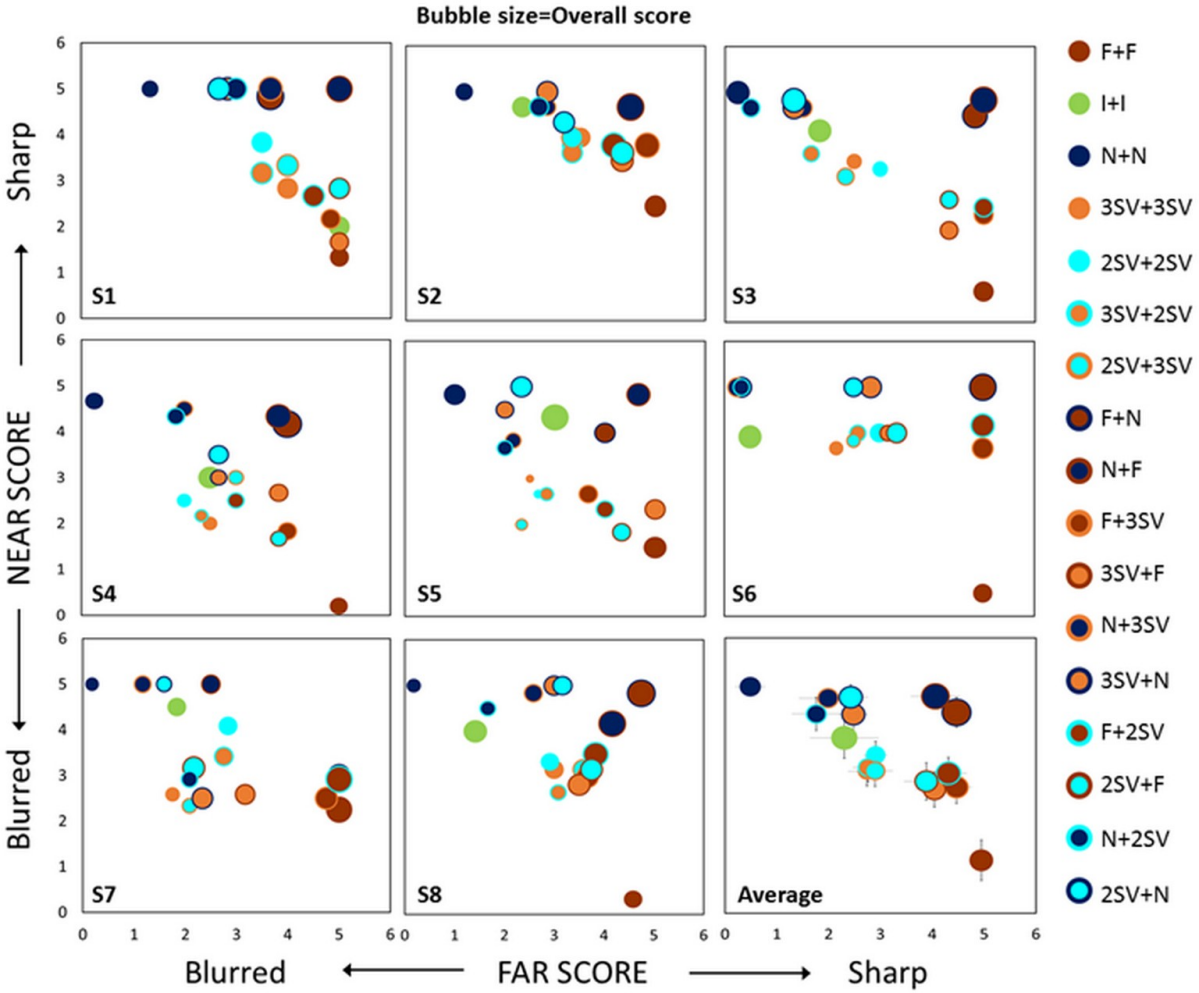
Binocular perceptual score for far (x-axis), near (y-axis) and overall (bubble size) for individual subjects (S1 to S8) and on average across subjects. The error bars (lower right box) represent the average PS variation across repetitions, averaged across subjects. They therefore show intersubject consistency in the scores, and not dispersion across subjects.

The PS for each subject is shown in Fig 4, where each box represents a different subject and each bubble represents a different presbyopic correction, with the position of the bubble indicating the perceptual score at far and near and the size indicating the overall score given to the visual scene.

The intersubject variabilities (differences across boxes) are manifest for all corrections. On the other hand, intra-subject repeatability of the scoring was consistent for all subjects, corrections and distances, with an overall average standard deviation of 0.45 PS, indicating that the differences in perceptual judgments found are subject-dependent and not due to measurement dispersion. The overall score (bubble size) was significantly higher (p<0.0001) for the Monovision and Modified Monovision corrections (3.8±1.2PS) than for binocular simultaneous vision corrections (2.5 ±0.5PS) on an average across subjects (left bottom panel). The overall score was highly correlated (p<10^−8^; R = 0.95; slope 1.02) with the average score across far, intermediate and near distances.

A multivariate ANOVA confirmed that the scores were significantly influenced by subjects (p<0.01, df = 7) and corrections (p<0.00001, df = 16). In addition, on average across conditions and subjects, the score was significantly lower for intermediate distance (2.64±1.04PS), than for far (3.11±1.36PS) or near (3.61±1.17 PS) distances (df = 2, p<0.0001). However there were no significant differences (p = 0.92) in PS when correcting the dominant eye for far or near (in Monovision and in Modified Monovision).

### Preferences to binocular presbyopic corrections

**Fig 5:**
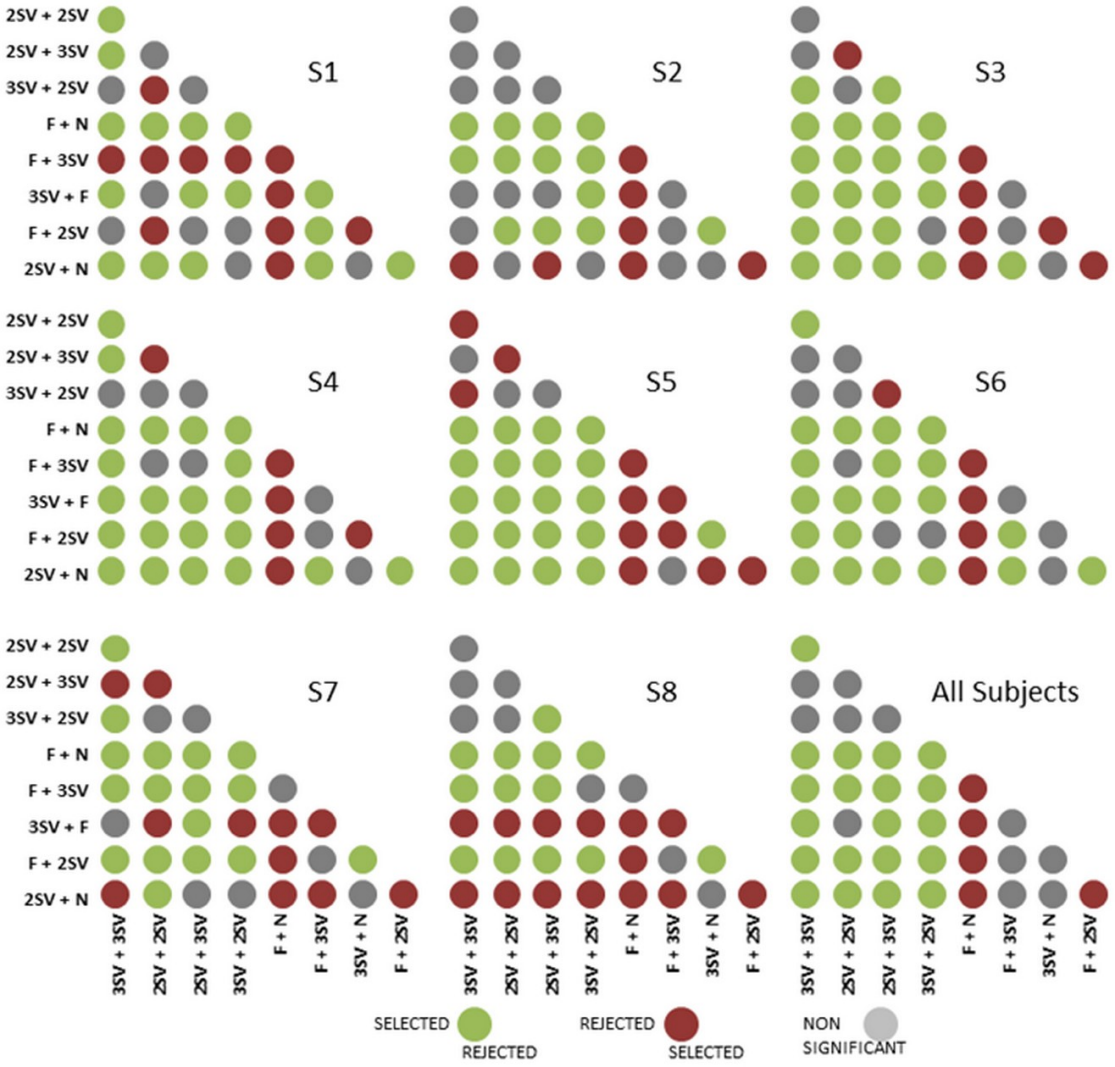
Preference maps for pair-wise comparison of binocular simultaneous vision, monovision and modified monovision corrections for all subjects (S1 to S8) and pooled across subjects.

Fig 5 shows the preference maps obtained from direct pairwise comparisons between presbyopic corrections, for each subject, and also averaged across all subjects. Green dots indicate that the correction represented on the y-axis was significantly preferred over the corresponding correction represented on the x-axis. Conversely, red dots indicate that the design on the y-axis was significantly rejected. A gray dot indicates non-significant preferences. The Monovision corrections were preferred 97% of the times and far-dominant Modified Monovision corrections (F+3SV or F+2SV) were preferred 63% of the times.

Similar to perceptual scoring, the binocular pattern preference also showed variations across subjects: subject S1 preferred near-dominant Modified Monovision correction and binocular bifocal simultaneous vision correction; subjects S2, S7, S8 preferred far dominant designs; and subjects S3-S6 preferred at least one monofocal correction compared to binocular simultaneous vision corrections. Interestingly, when all subjects are considered, the binocular bifocal (2SV+2SV) pattern was significantly preferred over any other binocular simultaneous vision corrections and the other simultaneous vision corrections were neither preferred nor rejected significantly (except binocular trifocal corrections).

## Discussion

Owing to increasing availability of new presbyopia correction options and new designs offering some advantages over conventional solutions [22], simultaneous vision corrections and combinations with Monovision are becoming increasingly popular [2, 13, 26, 27]. Simultaneous vision lenses provide fairly good visual quality regardless the viewing direction or distance, yet introduce a complex retinal blur [28, 29]. The ability of the subject to cope up with the new visual experience forms a key factor in the success of these presbyopic treatments. For these reasons, tools that enable patients to experience the blur introduced by simultaneous vision and monovision presbyopic corrections are needed both to further understand the modifications in vision produced by those corrections, and as clinical tool to optimize correction selections. In this work, we present a binocular, portable, see-through, wide field of view and programmable vision simulator that optically simulated various presbyopic corrections. Similar to our previous study examining multifocal vision applied monocularly [17], we demonstrated common trends in perceived visual quality with certain corrections, as well as strong individual differences, emphasizing the need of evaluating these corrections in every patient.

### Perceptual quality with binocular presbyopia corrections

Binocular monofocal corrections resulted in distance-dependent perceptual judgments. Intermediate monofocal correction provided good perceptual score in some subjects, however there was a large intersubject variability in the relative performance for near and far, indicating that under-correction as an option for presbyopia management is hard to customize across working distances or subjects. Also, the results from the simulations parallel reports from studies [3, 4, 30] that compare visual performance in eyes implanted with monofocal and with multifocal IOLs or contact lenses, which conclude that monofocal corrections outperform the multifocal intraocular lenses in both far and intermediate distances, but not at near.

Binocularly, simultaneous vision corrections produced a stable perceptual quality at far and near distances, with lower differences across subjects. The degradation in perceptual quality was not as severe as with monofocal corrections in certain conditions. In fact, none of the simultaneous vision corrections were perceived as blurred by the subjects, at any distance, suggesting that multifocal corrections provide a good compromise at all distances [31, 32].

Binocular perceptual quality with Monovision and Modified Monovision (far and near dominant) was excellent at far and near distances and fairly good at intermediate distances. Schor et al. [33] reported that both perceptual and binocular functions with Monovision corrections were better in pre-presbyopes than in presbyopes, although this may have been accounted by residual accommodation, that in our study was pharmacologically paralyzed. The perceptual outcomes with Monovision in our study could have been influenced by this age bias, as our subjects were primarily young subjects or pre-presbyopes (with artificially paralyzed accommodation). Some studies show that Modified Monovision corrections provide better binocular performance, greater comfort, and lesser glare compared to Monovision correction [13], yet in this study the perceptual quality in some patients for Modified Monovision was somewhat lower than that for Monovision.

### Ocular dominance vs sharpness dominance

In the current study, we found no differences in perceptual quality when the dominant eye or the non-dominant eye is corrected for far vision, suggesting that perception is driven by the presence of a sharp component. In a previous study in which simultaneous vision was simulated by image convolution we found that local sharpness dominated visual perception and adaptation [6]. Similarly, we found that when interocular differences in optical quality occurred, the sharpest eye drove adaptation [34]. Short term recalibration of blur perception was reported to occur binocularly, biased by eye with sharper retinal image and not independently for the two eyes when the retinal image quality was different between eyes [35] or in situations of binocular rivalry [36]. These findings explain why the perceptual quality was better with Monovision, irrespective of ocular dominance than with binocular simultaneous vision options. The current test did not considered assessing performance with 3-D targets, therefore not addressing conditions in which monovision corrections will likely underperform. A comprehensive visual assessment not restricted to perceptual quality, evaluating other meaningful aspects of visual experience with these corrections, such as stereopsis, comfort and binocular visual performance, may yield a different comparative ranking of binocular simultaneous vision and monovision corrections.

### Inter-subject differences

As in our previous study with monocular corrections [17], we found significant and systematic inter-subject variability in perceptual judgments. Here we found that perceptual quality was better with Monovision and Modified Monovision corrections in most subjects, yet these corrections were not equally judged by all subjects across different distances. These preferences were highly consistent within each subject. We speculate that the individual preferred correction may have been influenced by corrections that more closely represent the subject’s conventional visual experience, as suggested in previous studies on long-term adaptation to blur [34, 37, 38]. The intersubject differences further emphasize the need for a clinical instrument to simulate different presbyopic corrections before prescription, to customize the selection to the patient visual needs and perceptual preferences.

### Portable and binocular visual simulation

The current vision simulator represents an improvement from the hand-held monocular vision simulator [17] presented before, allowing now binocular simulation of presbyopic corrections. The presented simulator oriented to clinical use, as it features large field of view, is portable, see-through and functions well as a binocular system. While we have measured primarily binocular perceptual quality, the instrument can study binocular function such as stereopsis, which will add to a more comprehensive assessment of vision with the studied corrections. In fact, none of the subjects reported diplopia at any viewing distance throughout the measurements, which suggests that the instrument is well suited for measuring binocular motor functions with the presbyopic corrections.

We measured perceptual performance for generic simultaneous vision corrections. Tunable lenses have been shown to effectively simulate bifocals/multifocals of different patterns [6, 34, 35, 39]. Furthermore, tailoring of the through-focus optical quality of specific IOL designs, made possible by customizing the temporal coefficients in the multiplexing temporal pattern [40], allows representing of commercial real multifocal lenses and therefore providing the patient with a faithful simulation of post-operative vision. While the binocular vision simulator presented here is able to simulate the defocus halos due to multifocallity (created by light coming from planes that are out of focus), it does only provide an approximation to the anysometries in the image produced by segmented designs, or to the chromatic effects produced by diffraction designs, and does not reproduce the straylight produced by edge designs, nor surgical or biological aspects potentially affecting the final retinal image quality.

## Conclusions

In conclusion, we developed a custom-programmable binocular portable see-through vision simulator that can simulate different combinations of presbyopic corrections. Perceptual assessments using this binocular simultaneous vision simulator showed significant differences across subjects and corrections, with Monovision and Modified Monovision providing better perceptual quality when compared to other corrections.
